# Risk Factors for Breast Cancer in Third-Year Medical Students in NOVA Medical School

**DOI:** 10.7759/cureus.61814

**Published:** 2024-06-06

**Authors:** Mafalda Sanchez, Maria Castro, Miguel Rendeiro, Tomas Afonso, Ines Sancho, Joao Figueiredo, Zacharoula Sidiropoulou

**Affiliations:** 1 Surgery, NOVA Medical School, Lisbon, PRT; 2 Surgery, West Lisbon Local Health Unit EPE, Lisbon, PRT; 3 Breast Surgery, West Lisbon Local Health Unit EPE, Lisbon, PRT

**Keywords:** public health awareness, medical students, health literacy, breast cancer perception, risk factors for breast cancer

## Abstract

Comprehending the complexities of breast cancer, including its risk factors, methods for early detection, and treatment alternatives, is vital for effectively combating the illness and enhancing both survival rates and the quality of life for patients.

The current study, which is observational, descriptive, and cross-sectional in nature, was designed to assess the risk factors and perceptions related to breast cancer within a specific population. This research was carried out among third-year medical students at NOVA Medical School. The survey collected data on sociodemographic aspects and potential risk factors for developing breast cancer.

Results indicate that the sample consisted mainly of young females with a relatively low occurrence of known risk factors such as genetic predisposition and exposure to ionizing radiation. Analysis of the participants’ answers revealed a comprehensive understanding of recognized risk factors. Nonetheless, there was a divergence in views concerning the impact of body mass index before menopause.

This study underscores the importance of ongoing education regarding breast cancer and the factors that increase the risk of developing this disease.

## Introduction

Globally, breast cancer is the most frequently diagnosed malign neoplasm and it is responsible for more than two million cases per year. Moreover, it is the main cause of death by cancer in women worldwide [1].

Breast cancer is a heterogeneous disease, including multiple entities associated with distinct histologic and biological features, clinical presentation and behavior, and response to therapy [2].

Breast carcinoma in situ may present as ductal (also known as intraductal carcinoma) or as lobular carcinoma. This distinction is mainly based on the pattern of growth and the cytologic characteristics of the lesion.

Breast invasive carcinoma consists of many histological subtypes; the estimated percentage of each subtype, obtained by the analysis of a populational group of 135,157 women with breast cancer reported to the Surveillance, Epidemiology, and End Results (SEER) data bank of the North American Cancer Institute between 1992 and 2001: ductal infiltrative (76%); lobular invasive (8%); ductal/lobular invasive (7%); mucinous (2.4%); tubular (1.5%); medullar (1.2%); and papillary (1%). Other subtypes, including metaplastic breast cancer and micropapillary invasive breast cancer, are responsible for less than 5% of the cases [3].

Approximately half the cases of breast cancer are explained by known risk factors, such as reproductive factors and proliferative breast diseases. Another 10% are associated with family history and genetics of the individual. In addition, this risk can be modified by demographic, lifestyle, and environmental factors [4].

Breast cancer in stages I and II is usually treated with conservative surgery and radiotherapy. Radiotherapy after conservative surgery of the breast reduces mortality and recurrence. Stage III breast cancer usually requires neoadjuvant chemotherapy to reduce the size of the tumor and facilitate conservative breast surgery [5].

Understanding the nature of breast cancer, its risk factors, early detection methods, and treatment options is essential to effectively approach this disease and improve its survival rates and the patient’s quality of life.

## Materials and methods

Design and study population

This is an observational, descriptive, and cross-sectional study using an online questionnaire implemented between the 16th and the 28th of November 2023, conducted on a convenience sample that included a group of year three medical students from NOVA Medical School of Universidade Nova de Lisboa, in the year of 2023-2024. Our sample group included 120 students, out of which 90 responded, resulting in a total adhesion rate of 75%, while 86 fulfilled the inclusion criteria.

These students had access to electronic equipment, were informed about the study and its objectives, and consented to filling it completely.

Ethical considerations

This study was approved by the Ethics Committee of NOVA Medical School (116/2023/CEFCM) and the informed consent of the participants was obtained.

Questionnaire

A questionnaire was designed on Google Forms (based on references [6-10]) and conducted on the population under analysis as a tool to collect data from the participants. The questionnaire was disseminated through social media by the authors. Answers to the questionnaire were accepted in November 2023.

The questionnaire was divided into six main sections: Introduction; Inclusion criteria (which correspond to the second and third sections); Collection of sociodemographic data and prevalence of breast cancer risk factors; Assessment of the participant's perception of the variables that may or may not be considered risk factors for breast cancer; Registration of the participants’ email address (optional) for future contact if they are individuals at high risk of developing breast cancer [6-10].

Inclusion and exclusion criteria

The inclusion of participants in the study was based on the following criteria: consenting to participate in the study and knowing its purpose, being 18 years old or above, being a third-year medical school student, and having access to an electronic device: smartphone, computer, tablet, or other. The design of the questionnaire inherently served as an exclusion criterion by not permitting entries from individuals who did not fit these specific profiles.

Study variables

We analyzed a group of variables, which included age, sex, phototype, BMI, stature, personal history of benign breast disease, personal history of malignant breast disease, breast density, estrogen levels, androgen levels, use of hormonal substitution therapy, use of combined hormonal contraceptives, menarche age, menopause age, number of births, age of first pregnancy, radiation exposure, breastfeeding, family history of malignant breast disease, alcoholism, smoking habits, caffeine consumption, soy consumption, infertility, and previous pregnancy interruptions (spontaneous or voluntary).

Statistical analysis

Statistical analysis was initially carried out using Microsoft Excel^®^ software (Microsoft Corporation, Redmond, WA, USA) to organize and filter the data according to the inclusion and exclusion criteria. In a second instance, we used IBM SPSS Statistics^®^ software (v 22.0, IBM Corp., Armonk, NY, USA) for descriptive and inferential statistical analysis. The sociodemographic characteristics of the participants were described using frequencies (percentages) since the variables under study are categorical.

Qualitative variables, encompassing demographic characteristics, were elucidated using descriptive statistics while the chi-square test was employed to address the research inquiries using SPSS statistical software. P <0.05 defined statistical significance.

## Results

The study population (N=86) exhibited the following characteristics: sex distribution revealed 17 (19.8%) males and 69 (80.2%) females. In terms of age, the majority were in the 18-20 years range (65, 75.6%), followed by 21-23 years (19, 22.1%), and >23 years (2, 2.3%) with a mean age of 20.26 ± 1.02.

Phenotypically, 79 (91.9%) were Caucasian, 5 (5.8%) were Melanodermic, and 2 (2.3%) fell into the "Others" category.

Regarding weight, most of the participants were 51-60 kg (52, 60.5%), followed by 61-70 kg (18, 20.9%), 71-80 kg (8, 9.3%), >80 kg (5, 5.8%) and <50 kg (3, 3.5%) with a mean weight of 61.09 ± 10.27 kg. Most of the participants had a height of 1.61-1.70 m (51, 59.3%), followed by 1.70-1.80 m (17, 19.8%), >1.80 m (10, 11.6%), and 1.50-1.60 m (8, 9.3%) with a mean of 1.68 ± 0.08 m. The average BMI was 22.1, indicating a healthy weight range relative to height.

Menstruation onset occurred at 12-13 years (44, 51.2%), 14-15 years (13, 15.1%), 10-11 years (11, 12.8%), and >15 years (1, 1.2%). Regarding the use of oral contraceptives, 35 (40.7%) responded affirmatively and 51 (59.3%) negatively. Eighty two (98.3%) reported zero pregnancies, and 1 (1.2%) reported one.

Additionally, 8 (9.3%) had a first-degree relative with a history of breast cancer, 77 (89.5%) reported no tobacco habits, 6 (7%) were active smokers, and 3 (3.5%) were ex-smokers. Moreover, only 1 (1.2%) had a personal history of chest ionizing radiotherapy while 85 (98.8%) did not. All values were statistically significant (p<0.05) (Table 1).

**Table 1:** Demographic characteristics of participants

Characteristics	Frequency	Percentage	P-value
Sex			
Male	17	19.8	<0.05
Female	69	80.2	
Age (Years)			
18-20	65	75.6	<0.05
21-23	19	22.1	
>23	2	2.3	
Phenotype			
Caucasian	79	91.9	<0.05
Melanodermic	5	5.8	
Other	2	2.3	
Weight (Kg)			
<50	3	3.5	<0.05
51-60	52	60.5	
61-70	18	20.9	
71-80	8	9.3	
>80	5	5.8	
Height (m)			
1.50-1.60	8	9.3	<0.05
1.61-1.70	51	59.3	
1.70-1.80	17	19.8	
>1.80	10	11.6	
Menarche (years)			
10-11	11	12.8	<0.05
12-13	44	51.2	
14-15	13	15.1	
>15	1	1.2	
Use of anticonceptional pills			
Yes	35	40.7	0.08
No	51	59.3	
Number of pregnancies			
0	82	98.3	<0.05
1	1	1.2	
Age at the time of first pregnancy (years)			
<21	1	1.2	
Personal history of benign breast disease			
Yes	0	0.00	<0.05
No	86	100	
1^st^ degree relative with a history of breast cancer			
Yes	8	9.3	<0.05
No	78	90.7	
Alcohol habits			
Few times per month	47	54.7	<0.05
Few times per year	28	32.6	
Sometimes per week	2	2.3	
Never	9	10.5	
Tobacco habits			
Non-smoker	77	89.5	<0.05
Active smoker	6	7	
Ex-smoker	3	3.5	
Chest ionizing radiotherapy personal history			
Yes	1	1.2	<0.05
No	85	98.8	

Participants identified risk factors that can significantly (p<0.05) increase breast cancer risk, such as ionizing radiation exposure (100%), being female (98.8%), increasing age (94.2%), high BMI in menopause (81.4%), personal history of breast cancer (98.8%), high levels of estrogens (91.9%), a family history of breast cancer (96.5%), use of hormone replacement therapy in menopause (77.9%), early menarche (73.3%), or personal history of benign breast cancer (69.8%). Conversely, certain factors demonstrate a reduction in risk, such as regular physical exercise (86%) and breastfeeding (54.7%). Additionally, various risk factors exhibit inconclusive evidence or no influence such as 45.3% and 57% of participants indicated that high bone mineral density and caffeine intake have no influence on breast cancer. Moreover, according to the participant’s responses, most of the risk factors have an increasing effect on breast cancer except high suture, high bone mineral density, soja consumption, abortion, infertility, and caffeine intake. Breastfeeding and regular fascial exercise help reduce breast cancer, as indicated in Table 2.

**Table 2:** Risk factor distribution and association with risk perception of breast cancer

Risk factors	Risk variability	Frequency	Percentage	p-value	Most prevalent response
Female	Increase	85	98.8	<0.05	Increase
	No influence	1	1.2		
Age	Increase	81	94.2	<0.05	Increase
	No influence	3	3.5		
	Inconclusive evidence	2	2.3		
High BMI in menopause	Increase	70	81.4	<0.05	Increase
	No influence	10	11.6		
	Inconclusive evidence	6	7		
High BMI in pre-menopause	Increase	71	82.6	<0.05	Increase
	No influence	10	11.6		
	Inconclusive evidence	4	4.7		
	Reduce	1	1.2		
Tall stature	Increase	6	7	<0.05	No influence
	No influence	63	73.3		
	Inconclusive evidence	14	16.3		
	Reduce	2	2.3		
Personal history of benign breast disease	Increase	60	69.8	<0.05	Increase
	No influence	20	23.3		
	Inconclusive evidence	6	7		
Personal history of breast cancer	Increase	85	98.8	<0.05	Increase
	No influence	1	1.2		
High bone mineral density	Increase	21	24.4	<0.05	No influence
	No influence	39	45.3		
	Inconclusive evidence	13	15.1		
	Reduce	13	15.1		
High estrogen levels	Increase	79	91.9	<0.05	Increase
	No influence	2	2.3		
	Inconclusive evidence	2	2.3		
	Reduce	3	3.5		
Use of hormonal replacement therapy in menopause	Increase	67	77.9	<0.05	Increase
	No influence	2	2.3		
	Inconclusive evidence	4	4.7		
	Reduce	13	15.1		
Use of oral contraceptives	Increase	63	73.3	<0.05	Increase
	No influence	4	4.7		
	Inconclusive evidence	7	8.1		
	Reduce	12	14		
High level of androgens	Increase	36	41.9	<0.05	Increase
	No influence	26	30.2		
	Inconclusive evidence	12	14		
	Reduce	12	14		
Early menarche	Increase	63	73.3	<0.05	Increase
	No influence	11	12.8		
	Inconclusive evidence	7	8.1		
	Reduce	5	5.8		
Late menopause	Increase	56	65.1	<0.05	Increase
	No influence	13	15.1		
	Inconclusive evidence	4	4.7		
	Reduce	13	15.1		
Late first pregnancy	Increase	57	66.3	<0.05	Increase
	No influence	18	20.9		
	Inconclusive evidence	8	9.3		
	Reduce	3	3.5		
Nulliparity	Increase	49	57	<0.05	Increase
	No influence	18	20.9		
	Inconclusive evidence	5	5.8		
	Reduce	14	16.3		
Family history of breast cancer	Increase	83	96.5	<0.05	Increase
	No influence	3	3.5		
Alcoholism	Increase	65	75.6	<0.05	Increase
	No influence	14	16.3		
	Inconclusive evidence	7	8.1		
Tabagism	Increase	77	89.5	<0.05	Increase
	No influence	6	7		
	Inconclusive evidence	3	3.5		
Ionizing radiation exposure	Increase	86	100	<0.05	Increase
Breastfeeding	Increase	12	14	<0.05	Reduce
	No influence	22	25.6		
	Inconclusive evidence	5	5.8		
	Reduce	47	54.7		
Regular physical exercise	Increase	1	1.2	<0.05	Reduce
	No influence	8	9.3		
	Inconclusive evidence	3	3.5		
	Reduce	74	86		
Soy consumption	Increase	9	10.5	<0.05	No influence
	No influence	40	46.5		
	Inconclusive evidence	25	29.1		
	Reduce	12	14		
Infertility	Increase	22	25.6	<0.05	No influence
	No influence	34	39.5		
	Inconclusive evidence	19	22.1		
	Reduce	11	12.8		
Abortion	Increase (Rises)	26	30.2	<0.05	No influence
	No influence	45	52.3		
	Inconclusive evidence	11	12.8		
	Reduce	4	4.7		
Caffeine intake	Increase	16	18.6	<0.05	No influence
	No influence	49	57		
	Inconclusive evidence	17	19.8		
	Reduce	4	4.7		

## Discussion

The current study's findings align with the broader literature on breast cancer risk factors, emphasizing the multifactorial nature of the disease. For instance, a Mendelian randomization study suggests that type 2 diabetes mellitus may be causally associated with worse breast cancer-specific survival, indicating that managing diabetes could potentially improve prognosis [8]. Additionally, an extensive review of breast cancer epidemiology, risk factors, classification, and treatment strategies offers valuable information on the molecular subtypes of breast cancer and their implications for patient management [11]. These insights are particularly relevant to our study's demographic, which showed a low prevalence of traditional risk factors but a notable misperception regarding pre-menopausal body mass index as a risk factor.

Furthermore, the literature suggests that lifestyle and environmental factors play a significant role in breast cancer risk. A comprehensive review of these factors indicates that diet, physical activity, alcohol consumption, and environmental pollutants are all important considerations [12]. The study's findings on the participants' understanding of established risk factors, such as family history and radiation exposure, are corroborated by recent studies that analyze the role of germline CHEK2 variants and advocate personalized prevention strategies [13]. These studies highlight the importance of continuous education on breast cancer and its risk factors, reinforcing the necessity for comprehensive educational programs to ensure an accurate and current comprehension of breast cancer. This approach will enhance the capacity of healthcare professionals to effectively guide the population, promoting health and preventing breast cancer.

Detailed analysis of group risk factors

Gender

The group is predominantly female (80.2%), which is significant, as breast cancer is more common in females. P-value < 0.05 indicates that this is statistically significant.

Age

The majority of the group is between 18 and 20 years old (75.6%). Age is a significant risk factor for breast cancer, with the risk increasing with age. However, this group is relatively young, which generally suggests a lower risk.

Phenotype

The majority of the group is Caucasian (91.9%). Some research suggests that white women are slightly more likely to develop breast cancer than African American women.

Weight

The majority of the group weighs between 51 and 60 kg (60.5%). Being overweight or obese can increase the risk of breast cancer, especially after menopause. However, the weight range of this group doesn't suggest a high risk.

Height

The majority of the group is between 1.61 and 1.70 meters tall (59.3%). Some studies have found that taller women have a slightly higher risk of breast cancer.

Menarche

The majority had their first menstruation at 12-13 years old (51.2%). Women who started menstruating at an early age have a slightly higher risk of breast cancer.

Use of Contraceptive Pills

About 40.7% of the individuals use contraceptive pills. Some studies suggest a slight increase in breast cancer risk among women who use oral contraceptives. However, a p-value of 0.08 indicates that this might not be statistically significant.

Number of Pregnancies

Almost all individuals have not been pregnant (98.3%). Women who have not had a full-term pregnancy or have their first child after age 30 have a slightly higher risk of breast cancer.

Personal History of Benign Breast Disease

None of the individuals have a personal history of benign breast disease. Having certain types of benign breast lumps can increase the risk of getting breast cancer.

First-Degree Relative With a History of Breast Cancer

A small percentage (9.3%) have a first-degree relative with a history of breast cancer. Women with a first-degree relative (mother, sister, or daughter) who has had breast cancer have a higher risk.

Alcohol Habits

Most individuals consume alcohol a few times per month (54.7%). Alcohol consumption is clearly linked to an increased risk of developing breast cancer. The risk increases with the amount of alcohol consumed.

Tobacco Habits

Most individuals are non-smokers (89.5%). Many studies have found that breast cancer risk is higher among women who smoke compared to non-smokers.

Personal History of Chest Ionizing Radiotherapy 

Almost all individuals do not have a personal history of chest ionizing radiotherapy (98.8%). Women who had radiation therapy to the chest (including breasts) before age 30 are at an increased risk of breast cancer.

Please note that this comparison is based on the general consensus in the scientific community and individual risk can vary. For accurate information about breast cancer risk factors, it's always best to consult reliable medical sources or professionals. Also, remember that correlation does not imply causation, and these risk factors might be associated with the condition but do not necessarily cause it.

Analysis of individuals' perception regarding breast cancer risk factors

The risk factors most recognized by the students as increasing the risk of breast cancer (with a frequency over 80%) are ‘Female’, ‘Age’, ‘High BMI in menopause’, ‘High BMI in pre-menopause’, ‘Personal history of breast cancer’, ‘High levels of estrogens’, ‘Use of hormone replacement therapy in menopause’, ‘Use of oral contraceptive pills’, ‘Early menarche’, ‘Late menopause’, ‘Late first pregnancy’, ‘Tabagism’, and ‘Ionizing radiation exposure’. This suggests that these factors are well-known among the students.

The factors least recognized by the students as increasing the risk of breast cancer are ‘High stature’, ‘High bone mineral density’, ‘High level of androgens’, ‘Infertility’, and ‘Caffeine intake’. These factors might be less known or misunderstood among the students.

The factors that a significant number of students believe reduce the risk of breast cancer are ‘Breastfeeding’ and ‘Regular physical exercise’. While these factors are generally considered protective against breast cancer, it’s important to note that they don’t eliminate the risk.

Some factors show a spread of responses across ‘Increase’, ‘No influence’, ‘Inconclusive evidence’, and ‘Reduce’. These include ‘High bone mineral density’, ‘Use of hormone replacement therapy in menopause’, ‘Use of oral contraceptive pills’, ‘High level of androgens’, ‘Early menarche’, ‘Late menopause’, ‘Late first pregnancy’, ‘Nulliparity’, ‘Alcohol consumption’, ‘Tabagism’, ‘Breastfeeding’, ‘Regular physical exercise’, ‘Soja consumption’, ‘Infertility’, ‘Abortion’, and ‘Caffeine intake’. This suggests that there might be some confusion or lack of consensus among the students about these factors.

The factor that all students agreed increases the risk of breast cancer is ‘Ionizing radiation exposure’. This suggests a strong consensus on this factor.

By comparing the results obtained in this study with scientific consensus regarding possible breast cancer risk factors, which compiles information from several studies, we verified that the obtained responses in our study agree with the scientific consensus regarding the majority of the analyzed factors: female sex, age, high BMI in menopause, high stature, personal history of benign breast disease, personal history of breast cancer, high bone mineral density, high level of estrogens, use of hormone replacement therapy in menopause, use of contraceptive pills, high level of androgens, early menarche, late menopause, late first pregnancy, nulliparity, family history of breast cancer, alcohol consumption, tabagism, ionizing radiation exposure, breastfeeding, regular physical exercise, soy consumption, infertility, abortion, and caffeine intake.

We can divide these factors into three groups.

1. The group perception in the present study and the scientific consensus are in accord about these factors having the effect of increasing breast cancer risk: female gender, age, high BMI in menopause, personal history of benign breast disease, personal history of breast cancer, high levels of estrogens, use of hormone replacement therapy in menopause, use of contraceptive pills, early menarche, late menopause, late first pregnancy, nulliparity, familial risk of breast cancer, alcohol consumption, tabagism, and ionizing radiation exposure

2. The group perception in the present study and the scientific consensus are in accord about these factors having the effect of decreasing breast cancer risk: breastfeeding and regular physical exercise.

3. The group perception in the present study and the scientific consensus are in accord about the inconclusive evidence on the effect these factors have on breast cancer risk: high stature, high bone mineral density, high level of androgens, soy consumption, infertility, abortion, and caffeine intake.

On the other hand, there is a discrepancy in matters regarding the comparison between the influence of high premenopausal BMI in the present study and scientific consensus; this is the only factor where this discrepancy occurs. While in the present study, the group perception is that high premenopausal BMI contributes to an increased breast cancer risk, the scientific consensus is that evidence is inconclusive in this matter.

Investing in health literacy in medical students seems to be a good strategy to improve medical care since it will allow doctors to better advise patients and therefore, practice more preventive medicine [14]. It is also important to understand that health literacy is considerably multifactorial, as it is influenced by personal, cultural, and contextual factors [15]. Consequently, medical students should be aware of the need to target their interventions to every patient to maximize the effectiveness of every action.

Limitations

The study’s limitations include its reliance on a convenience sample of third-year medical students from NOVA Medical School, which may not accurately represent the general population, thus limiting the generalizability of the results. The cross-sectional nature of the study only allows for a snapshot in time and cannot establish causality. Data collection was based on self-reported information, which is susceptible to recall bias, and the selection criteria excluded individuals without access to electronic devices or social media, potentially introducing selection bias.

Furthermore, the study may not have included all relevant breast cancer risk factors, and the response rate was not reported, which could affect the result's validity. The statistical power of the study is unclear, without detailed participant numbers and prevalence rates. Participants’ higher awareness due to their medical education could skew results, and the cultural and educational context of the study may not reflect broader perceptions. Ethical considerations, such as confidentiality and data protection, were not discussed, which are crucial in human subject research. These points underscore the need for careful consideration when interpreting the study’s findings and suggest areas for improvement in future research.

## Conclusions

The study conducted at NOVA Medical School with medical students has shed light on the awareness and misconceptions surrounding breast cancer risk factors. Findings indicate a strong awareness among participants about well-known risk factors such as gender, age, and family history. However, there is a notable discrepancy in the understanding of the role of pre-menopausal body mass index, with the sample’s perception conflicting with scientific studies that find the association to be unclear. This highlights the need for ongoing education to align public perception with current scientific knowledge, ensuring healthcare professionals are well-equipped to guide and educate the community effectively.

Furthermore, the study underscores the value of health literacy among medical students, advocating for educational investments to enhance medical care and preventive medicine practices. Recognizing the multifaceted nature of health literacy, influenced by personal, cultural, and situational factors, the research suggests tailored educational interventions for each patient. This approach aims to optimize the impact of healthcare guidance and contribute to the broader goals of health promotion and breast cancer prevention.
